# Potato psyllids mount distinct gut responses against two different ‘*Candidatus* Liberibacter solanacearum’ haplotypes

**DOI:** 10.1371/journal.pone.0287396

**Published:** 2023-06-16

**Authors:** Xiao-Tian Tang, Julien Levy, Cecilia Tamborindeguy

**Affiliations:** 1 Department of Entomology, Texas A&M University, College Station, Texas, United States of America; 2 Department of Horticultural Sciences, Texas A&M University, College Station, Texas, United States of America; University of Saskatchewan College of Agriculture and Bioresources, CANADA

## Abstract

‘*Candidatus* Liberibacter solanacearum’ (Lso) is a bacterial pathogen infecting several crops and causing damaging diseases. Several Lso haplotypes have been identified. Among the seven haplotypes present in North America, LsoA and LsoB are transmitted by the potato psyllid, *Bactericera cockerelli* (Šulc), in a circulative and persistent manner. The gut, which is the first organ pathogen encounters, could be a barrier for Lso transmission. However, the molecular interactions between Lso and the psyllid vector at the gut interface remain largely unknown. In this study, we investigated the global transcriptional responses of the adult psyllid gut upon infection with two Lso haplotypes (LsoA and LsoB) using Illumina sequencing. The results showed that each haplotype triggers a unique transcriptional response, with most of the distinct genes elicited by the highly virulent LsoB. The differentially expressed genes were mainly associated with digestion and metabolism, stress response, immunity, detoxification as well as cell proliferation and epithelium renewal. Importantly, distinct immune pathways were triggered by LsoA and LsoB in the gut of the potato psyllid. The information in this study will provide an understanding of the molecular basis of the interactions between the potato psyllid gut and Lso, which may lead to the discovery of novel molecular targets for the control of these pathogens.

## Introduction

‘*Candidatus* Liberibacter solanacearum’ (Lso) is a Gram-negative and phloem-limited bacterium. Presently, at least 14 Lso haplotypes have been identified [1–11]. The haplotypes are transmitted by several psyllid species, and they infect different plants; some of which are important crops.

Haplotypes LsoA and LsoB are vectored by the potato psyllid (also known as the tomato psyllid), *Bactericera cockerelli* Šulc (Hemiptera: Triozidae) and infect solanaceous crops and wild plants. Specifically, both haplotypes are associated with potato zebra chip disease, which has been responsible for millions of dollars of losses in potato-producing regions of the United States, Mexico, Central America, and New Zealand [12–14]. They also affect the production of other important solanaceous crops such as tomato [15]. Difference in virulence between these two haplotypes was found in association with their host plants and their insect vector: in both cases LsoB was more pathogenic [16–21]. Currently, the most dominant control strategy available for the diseases associated with this pathogen is based on monitoring and managing psyllid populations by regular pesticide applications in an effort to limit the spread of Lso. However, frequent chemical applications negatively impact the environment and human health and contribute to the development of resistance in vector populations [22].

Disrupting the transmission cycle of pathogens within the insect vector is a novel and effective approach to control diseases. Lso is transmitted in a circulative and persistent manner by the potato psyllid [23,24]. Once ingested, Lso moves through the psyllid, from the gut lumen into the hemocoel and circulates to the salivary glands, from where it is disseminated to new host plants during psyllid feeding. Therefore, the gut represents the first significant barrier for the transmission of Lso by the psyllid after its ingestion. Indeed, the ability of Lso to infect the gut or to cross from the alimentary canal into the hemocoel could determine its transmission efficiency [25]. Thus, disruption of this process could be an effective way to control the spread of this pathogen.

Despite our adequate understanding of the invasion route of Lso within the potato psyllid, the mechanisms underpinning the transmission process remain largely unknown. In a previous analysis of LsoA and LsoB transmission, we determined that the titer of LsoB increases faster in the gut of adult psyllids than LsoA and that significantly higher LsoB titers were measured between 4 and 12 days of acquisition access period (AAP). Further, this difference in the acquisition was linked with a difference in transmission efficiency: 30 days after a 7-day AAP 90% of the test plants were positive for LsoB, while only 30% of the test plants were positive for LsoA [25]. In addition, the rates of Lso transmission also depend upon potato psyllid haplotypes, even with the same Lso haplotype [26].

Transcriptome analysis is a powerful methodology to quantify global gene expression patterns in various contexts from a single cell to whole tissues. Our earlier transcriptome studies analyzed the potato psyllid’s responses to Lso at the whole-body level, where an abundance of genes such as vitellogenin and heat shock proteins were differentially expressed [27]. That analysis also revealed that immune genes commonly induced in response to Gram-negative bacteria in other insects were absent [27]. Similar results were found in the Asian citrus psyllid, which is known to transmit a related Liberibacter species [28]. In addition, Ghosh et al. [29] found genes from the endoplasmic reticulum-associated degradation (ERAD) and the unfolded protein response (UPR) pathways overexpressed in the gut of carrot psyllid *B*. *trigonica* after infection with Lso haplotype D. Although the above studies have contributed to our understanding of the molecular basis of the interactions between Lso and its insect vectors, the transcriptional responses of the potato psyllid gut to the infection with LsoA and LsoB have not been evaluated. Considering our previous results that identified differences in the acquisition of these two haplotypes [25], evaluating the gut responses to each haplotype could be key to determine the molecular mechanisms involved in Lso acquisition. In turn, candidate genes to disrupt transmission could be identified.

Here, we took advantage of the existence of two bacterial haplotypes with different pathogenicity to identify key components involved in the gut responses to Lso based on transcriptome analyses. We compared the gut transcriptomes of potato psyllids after feeding on LsoA- or LsoB-infected tomato plants for 2 and 7 days, which correspond to early and late gut colonization times, respectively [25]. Our two objectives were to: (1) compare the psyllid gut responses to two Lso haplotypes—LsoA and LsoB—to identify the similarities and differences between these responses, and (2) provide insights into how the potato psyllid gut responds in a temporal manner to LsoA or LsoB. This study will contribute to the knowledge of the mechanisms underpinning the interactions between the potato psyllid and Lso at the gut interface, which can serve as a basis for developing new strategies to control vector-borne diseases based on pathogen transmission disruption.

## Materials and methods

All methods were performed in accordance with the relevant guidelines and regulations.

### Insects and plants

Moneymaker tomato plants were grown from seed (Victory Seed Company, Molalla, OR). The psyllid colonies (western haplotype) were maintained on tomato plants at room temperature 24 ± 1°C and photoperiod of 16: 8 h (L: D) in insect cages (24 × 13.5 × 13.5 cm, BioQuip, Compton, CA). LsoA- and LsoB-infected tomato plants were obtained by allowing psyllids from the LsoA- or LsoB-infected colonies to feed for one week on 6-week-old tomato plants [30]. Three weeks after insect infestation, the plants were tested for Lso infection using the LsoF/OI2 primers [31], the Lso haplotype was confirmed using the Lso SSR-1 primers [32].

### Lso infection, RNA purification and sequencing

Age-specific cohorts of female adult psyllids from the Lso-uninfected colony were collected and transferred to uninfected (control), LsoA- or LsoB-infected tomato plants for a 2- and a 7-day AAP. There were three replicates with 200 psyllid individuals for each AAP and Lso haplotype. The psyllids were collected from three independent colonies, and then transferred to three independent plants of each Lso haplotype. After infection, psyllid guts were dissected under the stereomicroscope (Olympus) as described in IbanezHancock and Tamborindeguy [33]. RNA samples for Illumina sequencing were purified using RNeasy Mini Kit (Qiagen, Hilden, Germany) followed by DNase I treatment with Turbo DNase (Ambion, Invitrogen, CA). Each RNA sample was ribo-depleted to remove psyllid rRNA using RiboMinus Transcriptome Isolation kit (Life Technologies, Carlsbad, CA) combined with 100 pmol of psyllid specific probes [34]. The depleted samples were submitted to Texas A&M AgriLife Genomics and Bioinformatic center for quality analysis, library preparation, and transcriptome sequencing. cDNA libraries were prepared using the TruSeq RNA Library Prep Kit v2 (Illumina®; San Diego, CA) following the manufacturer’s protocol. Libraries were multiplexed and sequenced on aHiseq-2500 platform (accession GSE206877).

### Bioinformatic analyses

The Illumina pipeline programs for sequence processing were used to produce FASTQ files, sort libraries and remove barcodes and adaptors. The high quality reads were analyzed in the Discovery Environment web interface and platform at CyVerse (http://www.cyverse.org/) using the Tuxedo suite [35]. The RNA-seq reads that passed the quality filters (i.e., Phred quality scores > 35) were mapped to the global transcriptome developed in our previous study [27]. The expression levels were calculated by using the Fragments Per Kilobase of transcript per Million mapped reads (FPKM) method. Genes with an adjusted *p*-value (false discovery rate, FDR) of less than 0.01 and fold change greater than or equal to 2.0 were considered as significantly differentially expressed genes (DEGs). RStudio (https://rstudio.com) was used for cluster dendrogram and principal component analyses (PCA). DEGs were annotated by BLASTx searches against NCBI’s non-redundant (nr) database (E-value cut off 1e-5) and UniProt database (https://www.uniprot.org/) using BLAST+ tools in Galaxy [36]. To further obtain an overview of the biological functions of the genes, all DEGs were subjected to Gene Ontology (GO) functional annotation using WEGO 2.0 (http://wego.genomics.org.cn/) [37] and mapped to terms in Kyoto Encyclopedia of Genes and Genomes (KEGG) pathway database using KOBAS 3.0 (http://kobas.cbi.pku.edu.cn/) and KEGG Mapper (https://www.genome.jp/kegg/tool/map_pathway2.html). Enrichment analysis was used to identify the GO terms and significantly regulated KEGG pathways.

### Real-time quantitative PCR (RT-qPCR) validation

Ten genes related to the representative category (e.g., digestion, ubiquitin, zinc finger, immunity, and mitochondria) were selected for validating the bioinformatic analyses by RT-qPCR. For this validation, female adult psyllids from the Lso-uninfected colony were allowed to feed for 2 and 7 days on uninfected, LsoA-, and LsoB-infected tomato plants as previously described to obtain independent samples (three replicates). The psyllids were collected from three independent colonies, and then transferred to three independent plants of each Lso haplotype. These samples were obtained independently of the samples used for RNAseq. RNA was purified from pools of psyllid guts as previously described. cDNA synthesis reactions were performed from each total RNA pool (independent from those used for sequencing). Five hundred nanograms of total RNA was reverse-transcribed into cDNA using Verso cDNA Synthesis kit (Thermo, Waltham, MA) and anchored-Oligo (dT) primers following the manufacturer’s instructions. The RT-qPCR reactions were performed using SensiFAST SYBR Hi-ROX Kit (Bioline, Taunton, MA) according to the manufacturer’s instructions. Each reaction contained 5 ng of cDNA, 250 nM of each primer (S1 Table) and 1X of SYBR Green Master Mix; the volume was adjusted to 10 μL using nuclease-free water. The RT-qPCR program was 95°C for 2 min followed by 40 cycles at 95°C for 5 sec and 60°C for 30 sec. RT-qPCR assays were performed using an Applied Biosystems™ QuantStudio™ 6 Flex Real-Time PCR System (Applied Biosystems). Reactions for all samples were performed in triplicates with a negative control in each run. The relative expression of the candidate genes were estimated with the delta delta CT method [38], using two reference genes of elongation factor-1a (GenBank KT185020) and ribosomal protein subunit 18 (GenBank KT279693) [39].

## Results

### Overview of the transcriptome dataset

We performed RNA-seq analysis of the gut of adult potato psyllids that were exposed to uninfected (control), LsoA- or LsoB-infected tomato plants for a 2- and a 7-day AAP, respectively. Therefore, six treatments were generated in this study: (1) 2 days Lso-free, (2) 7 days Lso-free, (3) 2 days LsoA-infection, (4) 7 days LsoA-infection, (5) 2 days LsoB-infection, and (6) 7 days LsoB-infection. Three biological replicates were analyzed per treatment, with a total of 18 independent libraries constructed. After sequencing, each library generated around 36–54 million reads and approximately 39.3–44.1% (S2 Table) of the reads in each library mapped to the global transcriptome developed in our previous study [27]. The PCA plot and cluster dendrogram of samples from the 18 libraries showed the mixture of non-infected and Lso-infected samples but a clear separation between the non-infected and the 2-day Lso-infected psyllids and 7-day Lso-infected psyllids based on the analysis of six treatments (S1 and S2 Figs).

**Fig 1:**
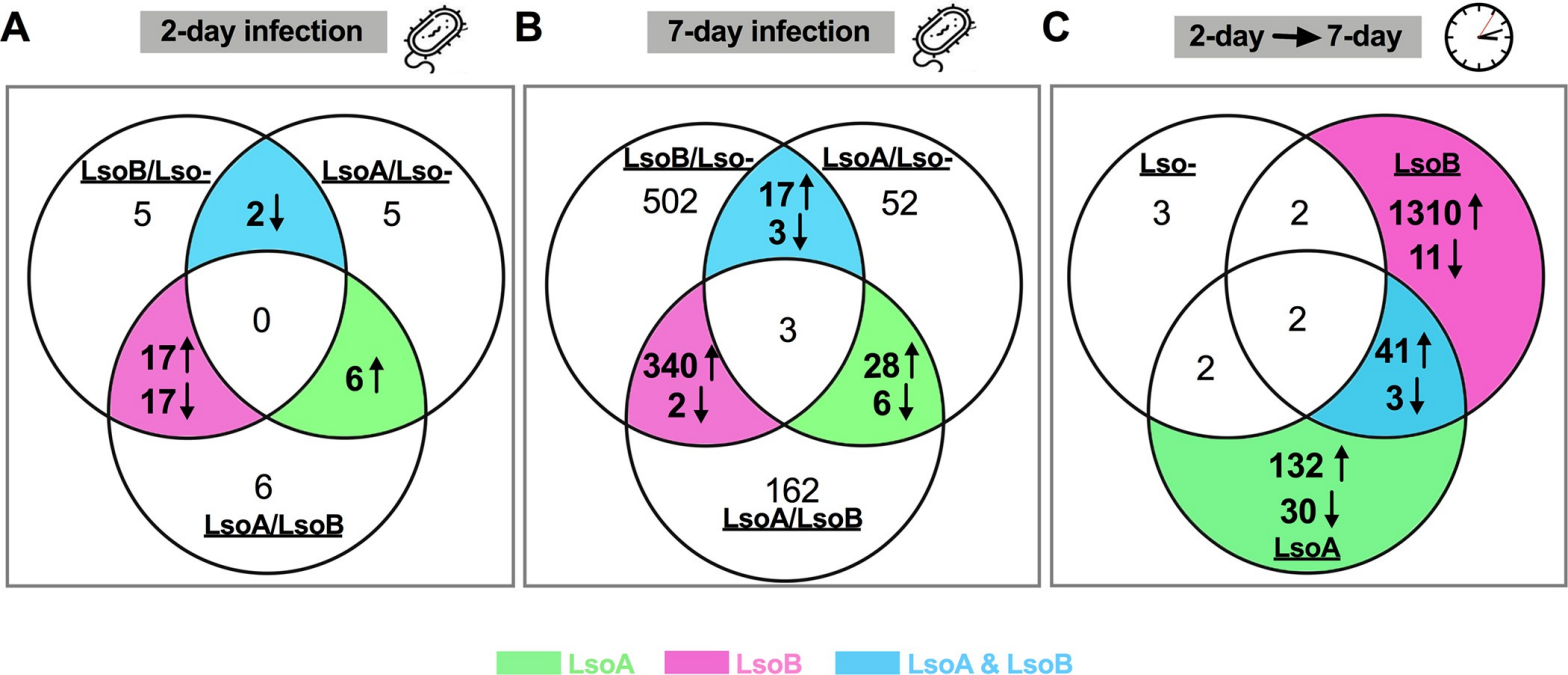
Venn diagram depicting unique and common DEGs in response to infection with LsoA or LsoB. (A) The number of DEGs in response to Lso after an AAP of 2 days; (B) The number of DEGs in response to Lso after an AAP of 7 days; (C) The number of DEGs in response to Lso after 2 days and 7 days. LsoA/Lso- indicates the comparison between LsoA-infected and Lso-uninfected psyllids. LsoB/Lso- indicates the comparison between LsoB-infected and Lso-uninfected psyllids. LsoA/LsoB indicates the comparison between LsoA- and LsoB-infected psyllids. The up arrow indicates up-regulation and the down arrow indicates down-regulation of DEGs.

A total of 1,580 DEGs were identified with an adjusted *p-*value (FDR) lower than 0.01 and a fold change greater than or equal to 2.0. There were 58 DEGs identified by comparing the gut transcriptomes of Lso-uninfected, LsoA-, or LsoB-infected insects after a 2-day feeding. Specifically, there were 41 DEGs when comparing the guts from the LsoB-infected and Lso-uninfected psyllids, 13 DEGs when comparing the guts from the LsoA-infected and Lso-uninfected psyllids, only 2 of those were common in these two comparisons, and both were down-regulated in response to the bacterial infection. Forty-six DEGs were identified when comparing the transcriptomes of the LsoA- and LsoB-infected guts. For the first objective of evaluating the haplotype effect, 34 DEGs with 17 up-regulated genes and 17 down-regulated genes were identified in response to LsoB specifically after a 2-day AAP; while only 6 DEGs were up-regulated in response to LsoA specifically. In addition, 2 DEGs were down-regulated in response to both LsoA and LsoB (Fig 1A).

More genes were differentially expressed in response to Lso after a 7-day AAP. A total of 1,115 DEGs were identified, of which 867 when comparing the gut transcriptomes of the LsoB-infected and Lso-uninfected psyllids, 109 when comparing the gut transcriptomes of the LsoA-infected and Lso-uninfected psyllids, and 541 when comparing the gut transcriptomes of the LsoA-infected and LsoB-infected psyllids. 342 DEGs with 340 up-regulated and 2 down-regulated genes were identified in response to LsoB specifically after an AAP of 7 days; while only 28 DEGs were up-regulated and 6 were down-regulated in response to LsoA specifically. In addition, 23 DEGs were identified in response to both LsoA and LsoB, and three of them were also differentially expressed when comparing the guts from the LsoA- and LsoB-infected psyllids (Fig 1B).

The temporal comparison of the gut transcriptomic profiles showed that there were 9 DEGs when comparing the guts of the Lso-uninfected psyllids, 210 DEGs when comparing the guts of the LsoA-infected psyllids, and 1,369 DEGs when comparing the guts of the LsoB-infected psyllids. Among those DEGs, 162 were exclusive to the response to LsoA with 30 down-regulated genes when comparing the 2- and 7-day AAPs, and 1,321 DEGs were only identified in response to LsoB with 11 of those being down-regulated. In addition, 44 DEGs with only 3 down-regulated genes were identified in response to both LsoA and LsoB (Fig 1C).

### DEGs associated with digestive proteins and transporters

Asian citrus psyllid gut digestive proteins such as aminopeptidase have been exploited by *Ca*. L. asiaticus” (CLas) [40]. In this study, we also found that several digestive enzymes such as carboxypeptidase, maltase, trehalase, lipase, aminopeptidase as well as proteases were up-regulated in response to LsoB specifically after an AAP of 7 days (Table 1). In addition to digestive enzymes, many genes encoding carbohydrate transporters such as facilitated trehalose transporter Tret1-like and glucose transporter were also up-regulated upon LsoB infection. Moreover, both LsoA and LsoB triggered the expression of ion transporters or channels involved in digestion; for example, sodium, potassium, zinc, heme as well as calcium channels displayed up-regulated profiles after an AAP of 7 days. Genes encoding ABC (ATP binding cassette) transporters also responded to LsoA and LsoB. These proteins transport endogenous substrates, hydrophobic compounds and metabolites across the plasma membrane (Table 1). However, no digestion-related genes were significantly regulated upon Lso infection after an AAP of 2 days.

**Table 1:** DEGs associated with gut digestion of potato psyllid.

	GI number	Annotation				Fold change
**Objective1: Haplotype**	**LsoA (7d)**			Free vs A/ A vs B		
	N/A	sodium channel protein type 2 subunit alpha-like				3.36/7.13
	gi|662225038	potassium channel subfamily K member 13-like, partial				3.31/3.53
	**LsoB (7d)**				Free vs B/ B vs A	
	gi|1041548206	carboxypeptidase D-like				6.57/-6.71
	gi|1060153063	maltase 2-like, partial				3.62/-3.74
	gi|662203161	trehalase isoform X3				4.76/-4.39
	gi|662187933	facilitated trehalose transporter Tret1-like				8.12/-6.98
	gi|662223811	facilitated trehalose transporter Tret1-like				5.53/-3.6
	gi|1041546106	LOW QUALITY PROTEIN: sodium leak channel non-selective protein-like				3.79/-3.64
	gi|1041537055	cyclic nucleotide-gated channel rod photoreceptor subunit alpha				4.6/-4.34
	gi|1036689888	sodium leak channel non-selective protein isoform X2				3.56/-4.38
	**LsoA&LsoB (7d)**		Free vs A/ Free vs B			
	gi|662187287	26S protease regulatory subunit 6A-B				7.04/9.78
**Objective2: Time**	**LsoA**					
	gi|1041530572	facilitated trehalose transporter Tret1-2 homolog				3.03
	gi|1041542154	facilitated trehalose transporter Tret1-like				3.05
	gi|1041545157	facilitated trehalose transporter Tret1-like				-2.72
	gi|1041552349	ABC transporter G family member 20-like				3.18
	gi|662225038	potassium channel subfamily K member 13-like, partial				5.88
	**LsoB**					
	gi|1101342295	cathepsin W-like				6.35
	gi|662185638	26S protease regulatory subunit 6A-B				6.37
	gi|1036066067	LOW QUALITY PROTEIN: aminopeptidase N-like				6.97
	gi|1101346724	glutamate carboxypeptidase 2-like				3.65
	gi|1041532450	lipase 1-like				3.65
	gi|662185638	inactive pancreatic lipase-related protein 1-like				6.37
	gi|662188926	maltase 1-like				6.03
	gi|1060153063	maltase 2-like, partial				5.02
	gi|662203161	trehalase isoform X3				4.11
	gi|1041551773	facilitated trehalose transporter Tret1-like				4.07
	gi|1041545133	facilitated trehalose transporter Tret1-2 homolog				4.41
	gi|662187933	facilitated trehalose transporter Tret1-like				10.01
	gi|662223811	facilitated trehalose transporter Tret1-like				8.02
	gi|662219141	facilitated trehalose transporter Tret1-like				4.86
	gi|1041534868	excitatory amino acid transporter-like, partial				3.62
	gi|662214523	equilibrative nucleoside transporter 3-like				4.36
	gi|1041541710	zinc transporter 1-like				5.11
	gi|1041551273	solute carrier organic anion transporter family member 5A1-like				2.62
	gi|1037058477	LOW QUALITY PROTEIN: glucose transporter type 1-like				5.48
	gi|662210441	heme transporter hrg1-B-like				5.06
	gi|1041540325	ABC transporter F family member 4-like				3.84
	gi|662220391	organic cation transporter protein-like, partial				3.87
	gi|662197848	cationic amino acid transporter 3-like				5.65
	gi|1041535278	proton-coupled folate transporter-like				5.33
	gi|1062687197	voltage-dependent calcium channel type A subunit alpha-1 isoform X39				3.12
	gi|662214997	two pore potassium channel protein sup-9-like				3.96
	gi|1041546106	LOW QUALITY PROTEIN: sodium leak channel non-selective protein-like				4.69
	gi|662192641	calcium channel flower				8.95
	gi|1041537055	cyclic nucleotide-gated channel rod photoreceptor subunit alpha				5.09

In objective1, for the LsoA category, the fold change values for the comparisons Lso-free vs. LsoA, and LsoB vs. LsoA are reported separated by the forward slash; for the LsoB category, the fold change values for the comparisons Lso-free vs. LsoB, and LsoB vs. LsoA are reported; and for the LsoA&LsoB category, the fold change values for the comparisons Lso-free vs. LsoA, and Lso-free vs. LsoB are reported. Negative values indicate down-regulation in the first treatment.

### DEGs associated with stress response, immunity, and detoxification

Microbial infection elicits immune responses and imparts stress. Several stress response-, immunity- and detoxification-related genes were regulated in response to both LsoA and LsoB but especially to LsoB after an AAP of 7 days rather than 2 days. The genes encoding heat shock protein 70 (HSP70) and glutathione S-transferases (GSTs) were up-regulated in response to LsoB after a 7-day AAP (Table 2). In addition, genes related to response to stress stimulus were also highly up-regulated, e.g., tribbles homolog 2, ras-related proteins, short neuropeptide F, GTP-binding protein, atrial natriuretic peptide receptor, and glutathione peroxidase (Table 2).

**Table 2:** DEGs associated with stress response, immunity, and detoxification of potato psyllid.

	GI number	Annotation	Fold change
**Objective1: Haplotype**	**LsoA(7d)**		
	gi|646706993	Laminin subunit alpha, partial	-5.77/-5.34
	gi|662224198	phosphoenolpyruvate carboxykinase, cytosolic [GTP]-like	4.74/5.07
	**LsoB (7d)**		
	gi|1041541888	baculoviral IAP repeat-containing protein 6-like	4.5/-4.76
	gi|1041533742	heat shock 70 kDa protein cognate 2-like	4.06/-3.7
	gi|1062672951	serine/threonine-protein kinase N isoform X7	5.58/-4.1
	gi|1314957697	serine/threonine-protein kinase PLK4	8.25/-9.27
	gi|1037109585	ETS-like protein pointed isoform X1	3.5/-3.24
	gi|1036052162	protein kinase C, brain isozyme isoform X3	4.38/-4.08
	gi|1062685985	nitrogen permease regulator 2-like protein	4.81/-3.89
	gi|31542100	tribbles homolog 2	4.96/-4.42
	gi|662218685	ras-related protein M-Ras-like	7.73/-7.33
	gi|662219677	short neuropeptide F-like, partial	4.72/-4.84
**Objective2: Time**	**LsoA**		
	gi|662196271	ras-related protein Rab-40C	2.66
	gi|662191295	probable phospholipid hydroperoxide glutathione peroxidase isoform X1	9.62
	gi|1060228182	laminin subunit alpha	-4.55
	gi|662224198	phosphoenolpyruvate carboxykinase, cytosolic [GTP]-like	5.89
	**LsoB**		
	gi|1101339243	autophagy-related protein 2 homolog B isoform X1	6.44
	gi|662185953	baculoviral IAP repeat-containing protein 5	7.41
	gi|1041541888	baculoviral IAP repeat-containing protein 6-like	8.81
	gi|1041533742	heat shock 70 kDa protein cognate 2-like	4.51
	gi|1041543694	glutathione S-transferase 1-like isoform X1	2.83
	gi|110456486	glutathione S-transferase-like protein, partial	4.7
	gi|1036955918	glutathione S-transferase D2-like	5.3
	gi|662189599	UDP-glucuronosyltransferase 2C1-like	7.19
	gi|1314957697	serine/threonine-protein kinase PLK4	5.87
	gi|1041531693	probable serine/threonine-protein kinase MARK-A	5.03
	gi|1062672951	serine/threonine-protein kinase N isoform X7	6.54
	gi|1041542874	serine/threonine-protein kinase SMG1-like	3.78
	gi|662225644	probable serine/threonine-protein kinase fhkE, partial	4.66
	gi|662194098	serine/threonine-protein kinase MARK2	4.38
	gi|662205647	microtubule-associated serine/threonine-protein kinase 3	5.16
	gi|1041543301	serine/threonine-protein kinase Genghis Khan	4.07
	gi|1037109585	ETS-like protein pointed isoform X1	3.86
	gi|1062685985	nitrogen permease regulator 2-like protein	4.68
	gi|662214654	ras-related GTP-binding protein A	5.3
	gi|58332432	tribbles homolog 2	7.34
	gi|662218685	ras-related protein M-Ras-like	11.61
	gi|662219677	short neuropeptide F-like, partial	6.48
	gi|1041532994	GTP-binding protein Rheb homolog	4.62
	gi|1041549075	atrial natriuretic peptide receptor 2-like	3.14
	gi|1041529912	low-density lipoprotein receptor-related protein 6	3.06
	gi|1041546276	mitogen-activated protein kinase kinase kinase kinase 5-like	5

In objective1, for the LsoA category, the fold change values for the comparisons Lso-free vs. LsoA, and LsoB vs. LsoA are reported separated by the forward slash; for the LsoB category, the fold change values for the comparisons Lso-free vs. LsoB, and LsoB vs. LsoA are reported. Negative values indicate down-regulation in the first treatment.

We also observed regulation of genes related to cellular and humoral immune responses. We found that whether from the infection type or the temporal perspective, after a 7-day AAP, LsoA down-regulated laminin subunit alpha and up-regulated phosphoenolpyruvate carboxykinase, which are involved in phosphatidylinositol 3-kinase (PI3K)—protein kinase B (Akt) signaling pathway and forkhead box O (FoxO) signaling pathway, respectively (Table 2). Genes involved in ubiquitin mediated proteolysis, apoptosis and autophagy pathways were up-regulated in response to LsoB after a 7-day AAP. For example, several ubiquitin-protein ligases, and genes encoding baculoviral IAP repeat-containing proteins (IAPs) and autophagy-related proteins (ATGs) were up-regulated in response to LsoB compared to Lso-free and LsoA (Tables 2 and 3). In addition, eight serine/threonine-protein kinase genes involved in signal transduction for numerous physiological events were induced specifically by LsoB after a 7-day AAP. The genes up-regulated by LsoB after a 7-day AAP included protein kinase C, nitrogen permease regulator 2-like protein and ras-related GTP-binding proteins, which are involved in the mechanistic target of rapamycin (mTOR) signaling pathway, and ETS-like proteins and mitogen-activated protein kinase (MAPK), which are involved in the MAPK signaling pathway (Table 2).

**Table 3:** DEGs involved in ubiquitin mediated proteolysis pathway.

	GI number	Annotation	Fold change
**Objective1: Haplotype**	**LsoB (7d)**		
	gi|1101399989	ubiquitin conjugation factor E4 A	6.18/-4.29
	gi|662221799	ubiquitin carboxyl-terminal hydrolase 15-like	3.67/-6.73
	gi|662213445	ubiquitin-fold modifier 1 isoform X1	3.51/-3.13
	gi|1062650979	(E3-independent) E2 ubiquitin-conjugating enzyme UBE2O	4.14/-3.67
	**LsoA&LsoB (7d)**		
	gi|1041552333	E3 ubiquitin-protein ligase TRIP12	4.48/3.16
**Objective2: Time**	**LsoA**		
	gi|1036784377	ubiquitin-conjugating enzyme E2 variant 2	3.68
	**LsoB**		
	gi|1041538933	mitochondrial ubiquitin ligase activator of nfkb 1-A-like	3.11
	gi|1101399989	ubiquitin conjugation factor E4 A	10.63
	gi|662221799	ubiquitin carboxyl-terminal hydrolase 15-like	9.57
	gi|662213445	ubiquitin-fold modifier 1 isoform X1	4.47
	gi|662185713	E3 ubiquitin-protein ligase RNF103-like	2.95
	gi|1060217161	ubiquitin-conjugating enzyme E2 W isoform X2	2.87
	gi|1062650979	(E3-independent) E2 ubiquitin-conjugating enzyme UBE2O	5
	gi|662212044	E3 ubiquitin-protein ligase HECTD1-like	4.81
	gi|662198106	E3 ubiquitin-protein ligase RNF180-like isoform X1	4.4
	gi|662197593	E3 ubiquitin-protein ligase UBR3	4.13
	gi|1314983068	E3 ubiquitin-protein ligase Nedd-4 isoform X9	7.64
	**LsoA&LsoB**		
	gi|1041552333	E3 ubiquitin-protein ligase TRIP12	7.63/7.8

In objective1, for the LsoB category, the fold change values for the comparisons Lso-free vs. LsoB, and LsoB vs. LsoA are reported separated by the forward slash; for the LsoA&LsoB category, the fold change values for the comparisons Lso-free vs. LsoA, and Lso-free vs. LsoB are reported. Negative values indicate down-regulation in the first treatment.

In addition to stress response and immunity pathways, LsoB also triggered detoxification pathways by up-regulating GSTs and UDP-glucuronosyltransferase genes (Table 2). The ABC transporters that are involved in detoxification in insects were up-regulated by LsoA and LsoB as well (Table 1).

### DEGs associated with gut epithelium renewal, cell cycle and DNA repair

It was shown that insect gut epithelium renewal is associated with pathogen infection [41]. In our study, we found that only LsoB induced the expression of genes involved in gut epithelium renewal, cell cycle and DNA repair after a 2- and 7-day AAP. First, the genes encoding armadillo segment polarity protein, protein kinase C, and protein wingless-like, which are involved in Wnt signaling pathway, as well as patj homolog and ras association domain-containing protein, which are involved in Hippo signaling pathway, showed up-regulated profiles after a 7-day AAP (Table 4). However, we also noted that after a 2-day AAP, LsoB down-regulated a wingless-like gene. Second, the genes related to cell proliferation processes such as cell cycle and repair were also up-regulated in response to LsoB after a 7-day AAP. For example, LsoB significantly induced the genes encoding ubiquitin-protein ligases, serine/threonine-protein kinase, sister chromatid cohesion protein, protein lin-52 homolog, general transcription factor IIH subunit as well as TATA element modulatory factor (Table 4).

**Table 4:** DEGs associated with cell renewal, cell cycle and DNA repair.

	GI number	Annotation	Fold change
**Objective1: Haplotype**	**LsoB (2d)**		
	gi|662221226	protein wingless-like	-17.56/23.58
	**LsoB (7d)**		
	gi|1060270998	armadillo segment polarity protein isoform X2	3.91/-3.98
	gi|1036052162	protein kinase C, brain isozyme isoform X3	4.38/-4.08
	gi|662221226	protein wingless-like	7.51/-6.65
	gi|662219483	cyclin-K isoform X1	10.6/-5.43
**Objective2: Time**	**LsoB**		
	gi|1101350861	ras association domain-containing protein 2 isoform X2	4.3
	gi|1189064107	patj homolog	6
	gi|662221226	protein wingless-like	12.96
	gi|1060270998	armadillo segment polarity protein isoform X2	5.02
	gi|1036950774	sister chromatid cohesion protein DCC1	4.42
	gi|662203336	protein lin-52 homolog	7.17
	gi|662209774	general transcription factor IIH subunit 4-like	5.16
	gi|1036716397	TATA element modulatory factor	3.85
	gi|1041548215	centrosomal protein of 97 kDa, partial	6
	gi|1698453	transposase	6.36
	gi|1101363356	GAS2-like protein pickled eggs	4.09
	gi|1041532257	transformation/transcription domain-associated protein-like	10.22
	gi|662219483	cyclin-K isoform X1	25.7

In objective1, for the LsoB category, the fold change values for the comparisons Lso-free vs. LsoB, and LsoB vs. LsoA are reported separated by the forward slash. Negative values indicate down-regulation in the first treatment.

#### DEGs associated with mitochondrial dysfunction and other functions

Intracellular pathogen infection modulates mitochondrial dynamics in vector cells [42]. In this study, a total of 21 mitochondrial proteins were differentially expressed in Lso-infected psyllid guts. Almost all of those genes were specifically up-regulated upon exposure to LsoB (Table 5). Two of those genes encoding pyruvate dehydrogenase and succinyl-CoA ligase, which are involved in the citric acid (TCA) cycle, were up-regulated after a 7-day AAP. In addition to the mitochondria-related genes, a large number of zinc finger genes involved in multiple functions (e.g., DNA binding) were significantly induced in response to LsoA and LsoB after a 7-day AAP (S3 Table). We also listed all the well-annotated down-regulated DEGs in S4 Table. For example, whether from the haplotype or the temporal perspective, after a 7-day AAP, LsoB down-regulated shootin-1 gene, which is involved in neuronal polarization and axon outgrowth. Some of these DEGs are uncharacterized proteins with unknown functions, which might also play key roles in the interactions between Lso and the potato psyllid (S4 Table).

**Table 5:** DEGs associated with mitochondrial function.

	GI number	Annotation	Fold change
**Objective1: Haplotype**	**LsoA (2d)**	** **	** **
	gi|662191692	HIG1 domain family member 2A, mitochondrial	30.98/150.18
	**LsoB (7d)**		
	gi|662212934	NADH dehydrogenase [ubiquinone] 1 alpha subcomplex subunit 10, mitochondrial	5.23/-8.9
	gi|164450052	mitochondrial ribosomal protein L12	3.4/-4
	gi|1041551426	pyruvate dehydrogenase [acetyl-transferring]-phosphatase 1, mitochondrial-like	4.01/-4.62
	gi|1036790856	ATP synthase subunit beta, mitochondrial	5.74/-3.4
	gi|662194219	succinyl-CoA ligase [ADP-forming] subunit beta, mitochondrial	4.08/-3.63
	**LsoA&LsoB (7d)**		
	gi|662191692	HIG1 domain family member 2A, mitochondrial	15.19/23.26
**Objective2: Time**	**LsoB**		
	gi|1041538933	mitochondrial ubiquitin ligase activator of nfkb 1-A-like	3.11
	gi|1041536900	mitochondrial import inner membrane translocase subunit Tim17-B-like isoform X1	5.32
	gi|662212934	NADH dehydrogenase [ubiquinone] 1 alpha subcomplex subunit 10, mitochondrial	7.17
	gi|662191692	HIG1 domain family member 2A, mitochondrial	36.96
	gi|662207693	glutamyl-tRNA(Gln) amidotransferase subunit C, mitochondrial	5.05
	gi|662185185	28S ribosomal protein S11, mitochondrial isoform X4	5.06
	gi|662214960	probable 39S ribosomal protein L45, mitochondrial	4.03
	gi|1041551426	pyruvate dehydrogenase [acetyl-transferring]-phosphatase 1, mitochondrial-like	4.84
	gi|662203079	28S ribosomal protein S15, mitochondrial	3.64
	gi|662187983	D-beta-hydroxybutyrate dehydrogenase, mitochondrial	4.63
	gi|662206948	28S ribosomal protein S33, mitochondrial	4.2
	gi|1041544248	aldehyde dehydrogenase, mitochondrial	4.69
	gi|1006107229	Lipoamide acyltransferase component of branched-chain alpha-keto acid dehydrogenase complex, mitochondrial	3.61
	gi|1060142930	mitochondrial chaperone BCS1	5.92
	gi|1036790856	ATP synthase subunit beta, mitochondrial	5.12
	gi|662189998	mitochondrial fission process protein 1	4.79
	gi|662194219	succinyl-CoA ligase [ADP-forming] subunit beta, mitochondrial	3.49
	gi|1041543152	rRNA methyltransferase 2, mitochondrial isoform X2	4.02
	gi|1036769629	mitochondrial cardiolipin hydrolase-like	6.57
	gi|662190957	39S ribosomal protein L30, mitochondrial	-3.57

In objective1, for the LsoA category, the fold change values for the comparisons Lso-free vs. LsoA, and LsoB vs. LsoA are reported separated by the forward slash; for the LsoB category, the fold change values for the comparisons Lso-free vs. LsoB, and LsoB vs. LsoA are reported; for the LsoA&LsoB category, the fold change values for the comparisons Lso-free vs. LsoA, and Lso-free vs. LsoB are reported. Negative values indicate down-regulation in the first treatment.

### Gene Ontology (GO) annotation and Kyoto Encyclopedia of Genes and Genomes (KEGG) pathway

**Fig 2:**
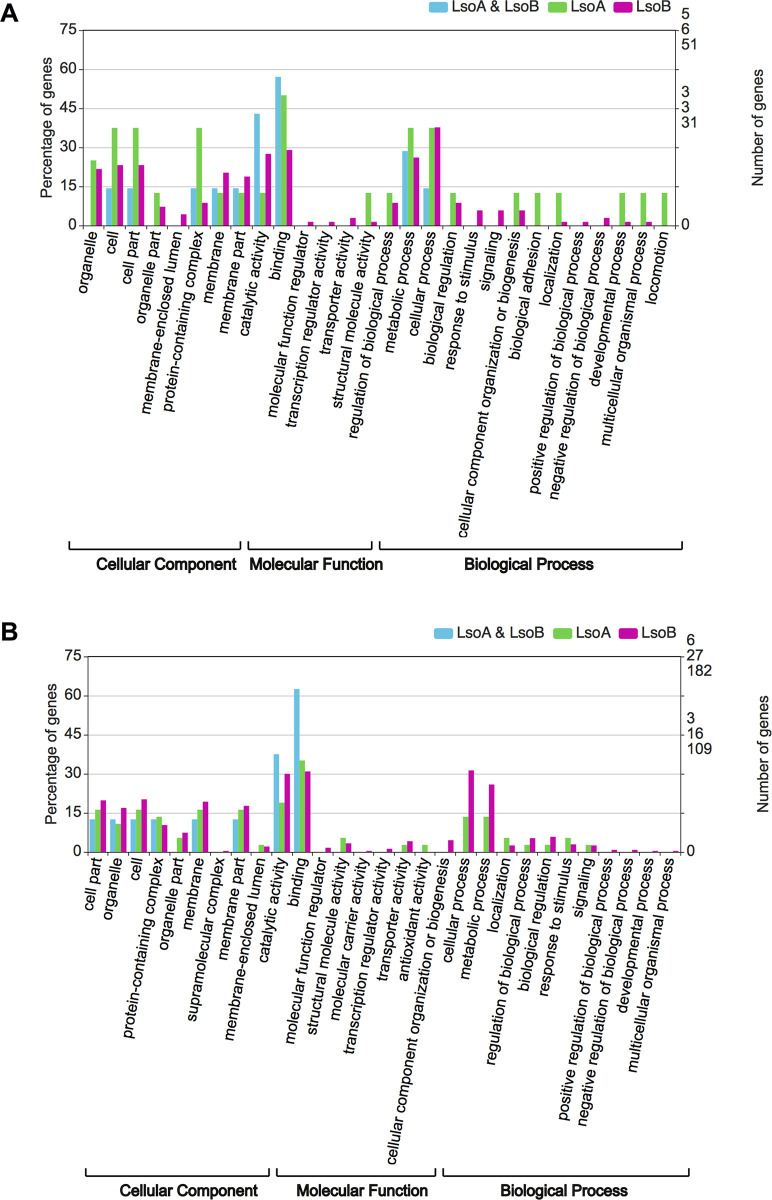
GO enrichment analysis. (A) GO classification of DEGs based on the first objective: The haplotype effect; (B) GO classification of DEGs based on the second objective: The temporal profile (from 2 days to 7 days). The GO annotations are separated into the “Cellular Component”, “Molecular Function”, and “Biological Process” categories.

**Fig 3:**
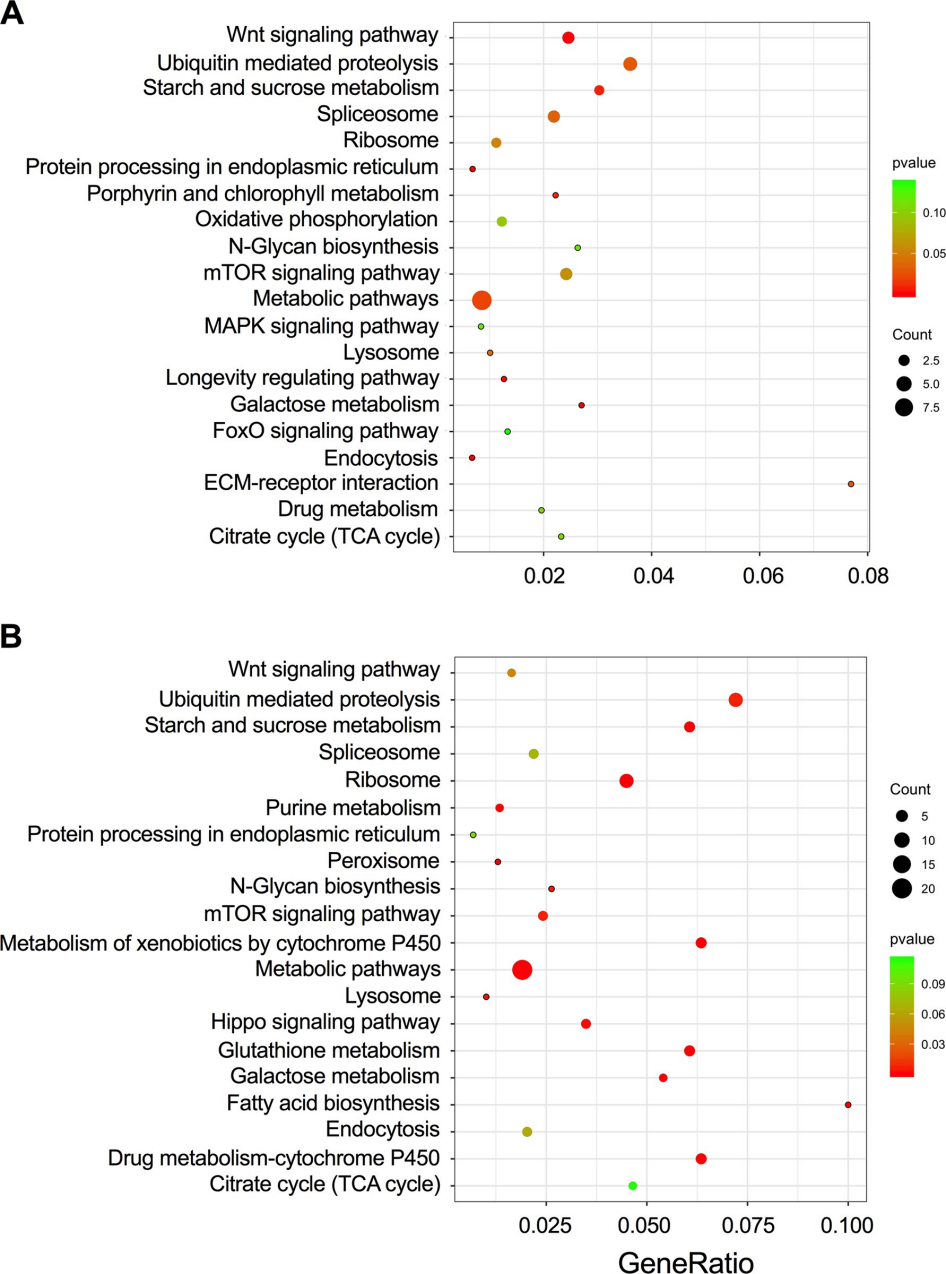
KEGG pathway analyses of DEGs in response to LsoB. (A) The top 20 significant enriched pathways based on the first objective: The haplotype effect (for both 2 days and 7 days’ infection); (B) The top 20 significant enriched pathways based on the second objective: The temporal profile (from 2 days to 7 days). Y-axis label represents pathway and X-axis label represents enrichment factor.

To identify the biological roles of the DEGs, we further utilized GO enrichment analyses to determine the functions of the DEGs. From the perspective of the haplotype effect (for both 2- and 7-day infection), the DEGs were categorized into 28 secondary GO categories under the cellular component, molecular function, and biological process divisions. Specifically, both LsoA and LsoB mostly induced genes with “catalytic activity”, “binding”, “metabolic process”, and “cellular process” annotations. However, the DEGs with the “biological adhesion” and “locomotion” annotations were exclusive to the response to LsoA while LsoB mostly changed the expression of genes in the “membrane-enclosed lumen”, “response to stimulus”, and “signaling” categories (Fig 2A). From the temporal perspective, the enrichments of DEGs were largely similar to the assignments from the haplotype perspective with “catalytic activity”, “binding”, “metabolic process”, and “cellular process” as the most represented terms. However, only LsoA infection mostly changed the expression of genes with “antioxidant activity”; genes in the categories such as “cellular component organization or biogenesis” and “molecular function regulator” were in regulated response to LsoB specifically (Fig 2B). To identify signaling pathways involved in Lso and potato psyllid interactions at the gut interface, we mapped the DEGs to the KEGG database. The top 20 enriched pathways in response to LsoB are shown in Fig 3. From the haplotype effect perspective (both for 2- and 7-day infection), the DEGs were highly clustered in the “Metabolic”, “Ubiquitin mediated proteolysis”, “Wnt signaling pathway” and “mTOR signaling” pathway (Fig 3A). From the temporal perspective, the “Metabolic”, “Ubiquitin mediated proteolysis”, “Hippo signaling pathway”, and “Ribosome” were the main regulated pathways (Fig 3B). However, few enriched pathways such as metabolic and ubiquitin-mediated pathways were identified for the DEGs in response to LsoA, possibly due to the lower number of genes significantly induced or repressed.

### Validation and expression profiles of DEGs

**Fig 4:**
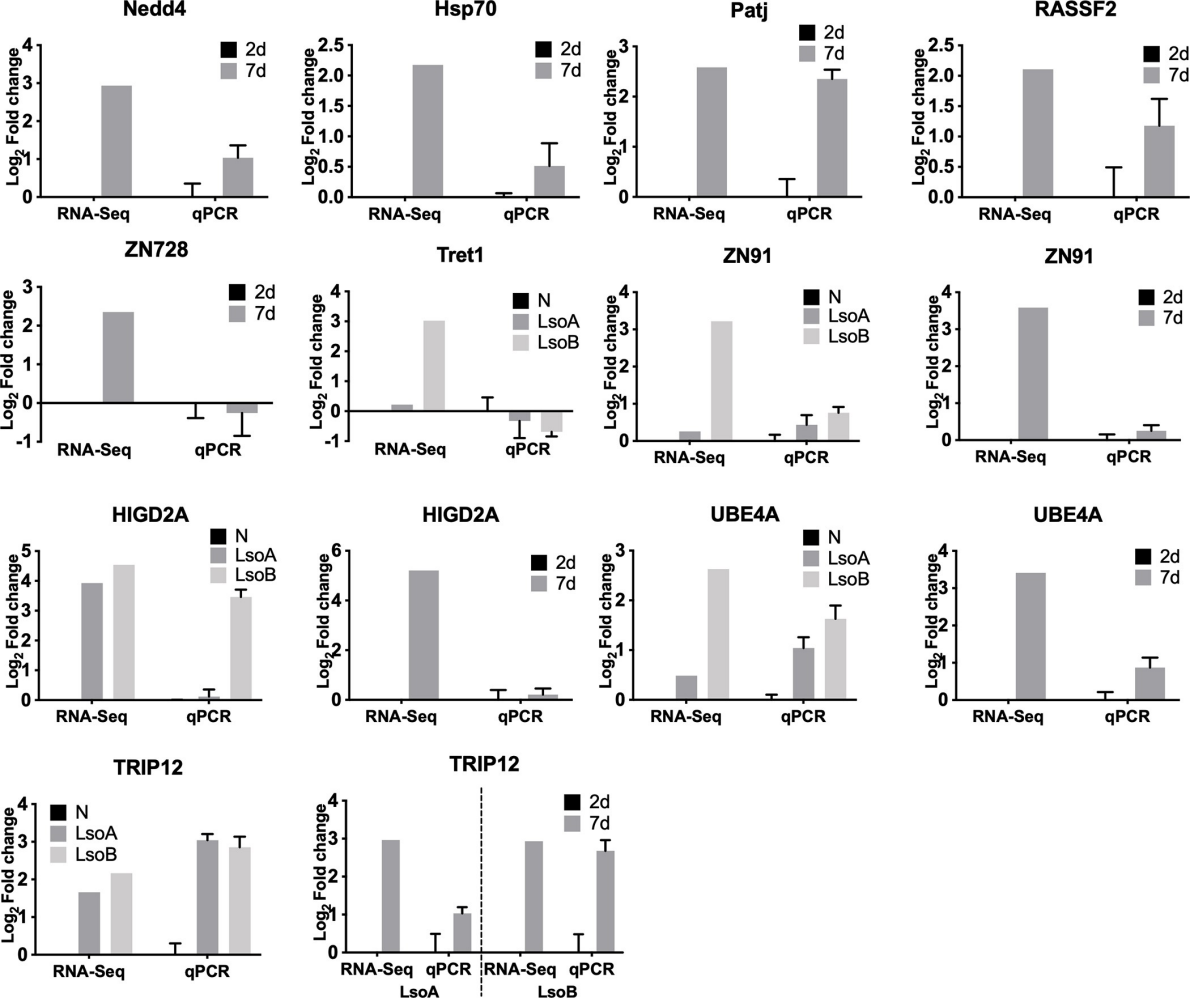
Comparison of gene expression patterns obtained by RNA-Seq and RT-qPCR. The mean ± SE was calculated to determine the relative transcript levels using the delta delta CT method.

To validate the accuracy and reproducibility of the transcriptome bioinformatic analyses, ten DEGs related to digestion, ubiquitin, immunity, mitochondrial proteins, cell repair and zinc finger proteins were selected for qPCR verification. The ten genes included E3 ubiquitin-protein ligase Nedd-4 isoform X9 (Nedd4); E3 ubiquitin-protein ligase TRIP12 (TRIP12); heat shock 70 kDa protein cognate 2-like (Hsp70); patj homolog (Patj); ras association domain-containing protein 2 isoform X2 (RASSF2); zinc finger protein 728-like (ZN728); zinc finger protein 91 (ZN91); facilitated trehalose transporter Tret1 (Tret1); HIG1 domain family member 2A; mitochondrial (HIGD2A); and ubiquitin conjugation factor E4 A (UBE4A). The qPCR validation was performed using independent samples from the ones submitted for sequencing. We found that most of the genes showed concordant direction of change between the bioinformatic and qPCR results except for two genes, ZN728 and Tret1 (Fig 4); indicating the accuracy and reliability of our DGEs libraries.

## Discussion

We have previously shown differences in the accumulation of LsoA and LsoB in the gut of adult psyllids, which correlated with difference in their transmission [25]. The analysis of the gut transcriptome can reveal the potential mechanisms underlying the molecular interactions between the potato psyllid and Lso.

In general, we found that each Lso haplotype triggered a unique transcriptional response, with most of the distinct genes elicited by LsoB. Indeed, results from our previous studies indicated that LsoB was more pathogenic to its host plants and insect vector [16–20]. The differences in pathogenicity could be linked to specific genes, for example distinct effector proteins secreted by each Lso haplotype. Meanwhile, the Lso copy number could also contribute to the difference of gene expression since our previous studies showing LsoA copy number is lower than that of LsoB in the gut [25]. It is also noteworthy that most DEGs were up-regulated by Lso infection, while only few were down-regulated, which contrasts with the gut transcriptome analysis of the Asian citrus psyllid in response to CLas [43], in which most of the DEGs were down-regulated. Differences in the response of adult psyllids to CLas and Lso are expected. Several differences have been described between these two pathosystems: while CLas infection increases the fecundity of Asian citrus psyllid females [44], Lso infection results in decreased fecundity [45,46], or psyllids that acquire CLas as adults rarely vector this pathogen [47–49], while potato psyllids can efficiently acquire and transmit Lso as adults [25,50]. These differences between these systems could be linked to differences between the insects, for instance immune responses elicited, or differences between the pathogens such as distinct secreted effectors. Discrepancies between the two systems were already described through transcriptome analyses [51] or the evaluation of programmed cell death in the gut of the vectors in response to the pathogens [52,53]. Furthermore, the gut response of the carrot psyllid *B*. *trigonica* gut to Lso was also different, as many key genes involved in the ERAD and UPR pathways in were induced by Lso haplotype D [29] but not in the present study.

Lso can alter the physiology of its vector [54], but the physiology and molecular biology of the potato psyllid digestive system has rarely been reported. Exposure to CLas repressed several Asian citrus psyllid digestive enzymes and the expression of transporters [43]. Our transcriptome analysis revealed that LsoB regulated the expression of genes encoding all the common insect digestive enzymes, carbohydrases, lipases, and proteinases after a 7-day AAP. Digestive proteases, which include serine proteases (e.g., trypsin and chymotrypsin) and cysteine proteases (e.g., cathepsin), are expressed abundantly in some hemipteran insects such as aphids and planthoppers [55–57]. None of the serine proteases were induced by LsoB; however, cathepsins, which often are active at slightly acidic pH [58], were up-regulated. It is interesting that none of the genes encoding digestive enzymes were regulated by LsoA. However, both LsoA and LsoB up-regulated sugar transporters and ion channels such as the facilitated trehalose transporters and potassium channels. Trehalose is the most abundant sugar in the insect hemolymph, and is the main source of energy and carbon for insects. It has been observed that changes in the membrane environment of cells alter the activity and metabolism of trehalose [59]. Based on our study, it is likely that the regulation of trehalose transporters by Lso changes the energy uptake or even the physiology of the potato psyllid gut and hemolymph. This phenomenon was also observed in the Asian citrus psyllid, however in this latter species facilitated trehalose transporters were down-regulated by CLas [43]. Lso could also disturb ion homeostasis in the psyllid gut by up-regulating ion transporters or channels. For example, ABC transporters import or export a wide variety of substrates ranging from small ions to macromolecules [60]. They are mainly involved in diverse cellular processes such as the maintenance of the osmotic homeostasis, nutrient uptake, immunity against bacteria and pathogenesis [60,61]. In our study, several ABC transporters were among the DEGs in response to LsoA and LsoB. Overall, Lso appears to affect psyllid digestion at the later infection period since none of the digestion-related genes were regulated significantly upon Lso infection after a 2-day AAP.

In addition, Lso might modify psyllid metabolic processes based on the evidence that LsoA and LsoB infection resulted in the regulation of genes annotated within the “metabolic process” of the biological process category and “metabolic” signaling pathways based on GO and KEGG enrichment analyses. It has been shown previously that liberibacter bacteria can manipulate their insect vectors’ energy metabolism. For example, CLas alters the energy metabolism of its vector securing its need for energetic nucleotides [62]. Besides, we also observed that Lso regulated many genes involved in mitochondrial functions and the TCA cycle. For example, three key enzymes—pyruvate dehydrogenase, isocitrate dehydrogenase and succinyl-CoA ligase—were up-regulated by LsoB; however, these genes were down-regulated in the Asian citrus psyllid by CLas [43], which the same pattern as observed for the digestion enzymes.

**Fig 5:**
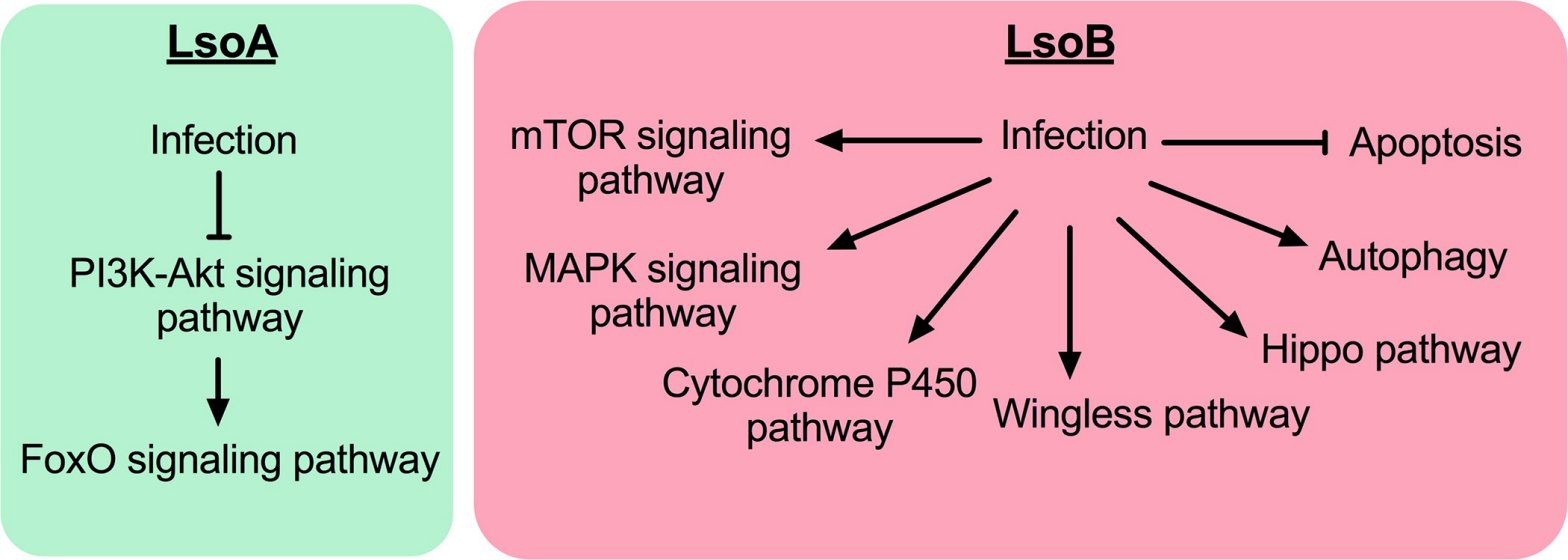
Distinct immune pathways were triggered by LsoA and LsoB in the gut of potato psyllid.

The innate immune response plays a key role in the defense against microbial infections in invertebrates [63]. Our data showed that following Lso infection, the expression of genes involved in the psyllid immune responses including the humoral and cellular responses were induced. The activation of immune responses is probably the evolved strategy of the potato psyllid to protect itself to some extent from the deleterious effects of Lso. It is interesting that LsoA and LsoB seem to induce different immune pathways (Fig 5) in the potato psyllid gut. Specifically, LsoA repressed the PI3K-Akt pathway but activated the FoxO signaling pathway by down-regulating laminin subunit gene and up-regulating phosphoenolpyruvate carboxykinase. In fact, the FoxO transcription factors are negatively regulated by the PI3K/Akt signaling pathway and considered to have an inhibitory effect on cell proliferation and survival [64,65]. Therefore, it is most likely that LsoA promotes gut cell death by inhibiting the PI3K-Akt pathway and activating FoxO signaling pathway. In contrast, we observed that two inhibitors of apoptosis were up-regulated by LsoB after an AAP of 7 days. Recently, programmed cell death (apoptosis and autophagy) gained importance as affecting the transmission of plant pathogens by phloem-feeders such as whiteflies, planthoppers and leafhoppers [66–68]. We hypothesize that the regulation of the cell death pathways could be a psyllid adaptation to limit Lso invasion or a strategy used by Lso to enhance its transmission. Apoptosis was reported in the gut of CLas-infected Asian citrus psyllid adults [52] but no evidence of apoptosis was found in the gut of nymphs [69]; this response could be a factor explaining the developmental differences of CLas acquisition by the vector. In our previous studies, we found no evidence of apoptosis in the gut of potato psyllid adults [53] and that both LsoA and LsoB up-regulated inhibitors of apoptosis [70] and autophagy [71]. In the present study, we also found genes involved in mTOR and MAPK pathways specifically up-regulated by LsoB after an AAP of 7 days. Both pathways play important roles in energy homeostasis and innate immunity, regulating cell proliferation and survival [72–74].

LsoB also triggered detoxification pathways by up-regulating GSTs and UDP-glucuronosyltransferase genes. GSTs comprise a diverse class of enzymes that detoxify stress-causing agents, including toxic oxygen free radical species, and they can be up-regulated in arthropods upon oxidative stress and microbial challenges [75,76]. In the Asian citrus psyllid gut, cytochrome P450s, GSTs and UDP-glucuronosyltransferase were also triggered by CLas but showed decreased expression profiles [43]. As indicated above, ABC transporters which are also involved in detoxification [77] were up-regulated by Lso as well. This suggests that these two liberibacter species have distinct effects on their vectors.

The ability of the host to survive an infection relies not only on resistance mechanisms that eliminate the pathogen but also on tolerance mechanisms that increase the capacity of the host to endure the infection [78]. The function of gut epithelial renewal in host-pathogen interactions has been well demonstrated. For example, Buchon et al. [41] observed that ingestion of the bacterium *Erwinia carotovora* subsp. *carotovora* 15 provokes a massive increase in *Drosophila* epithelial renewal. In our study, we found that the genes or pathways involved in gut epithelium renewal, cell cycle and DNA repair were exclusively induced by the highly virulent LsoB after an AAP of 7 days. Probably, genes such as protein wingless-like and patj homolog in Wnt, as well as those involved in the Hippo signaling pathway may help attenuate the gut cell damage by LsoB infection. We also noted that in response to LsoB, the expression of a wingless-like gene was reduced after an AAP of 2 days, just when LsoB is about to enter the gut cells. In addition, some genes from the ubiquitin-proteasome system play roles in regulating the Hippo pathway. For example, E3 ubiquitin-protein ligase Nedd4 works as a key regulator of the Hippo signaling pathway and overexpression of this gene in *D*. *melanogaster* induces proliferation of the midgut epithelium [79]. In our study, the Nedd4 gene was also up-regulated by LsoB after an AAP of 7 days. Additionally, it has been demonstrated that pathogens are able to alter the host cell cycle to achieve the replication and expression of their genomes [80]. The up-regulation of genes such as serine/threonine-protein kinase and sister chromatid cohesion protein suggests that LsoB probably disturbs the normal cell cycle and DNA repair in the potato psyllid gut [81,82].

In summary, ingestion of Lso had a dramatic impact on the physiology of psyllid gut that included the modulation of the expression of genes involved in digestion and metabolism, stress response, immunity, detoxification as well as cell proliferation and epithelium renewal. Furthermore, our data showed that the potato psyllid mounts distinct responses upon infection with two Lso haplotypes from both the haplotype and temporal perspectives. Few genes were regulated after an AAP of 2 days, but after an AAP of 7 days the majority of DEGs were involved in digestive function, immunity, cell renewal, and mitochondrial function. The information in our study may offer hints for discovery of novel and specific molecular targets for disrupting Lso transmission within psyllid vector.

## Supporting information

S1 Fig Principal component analysis (PCA) of 18 libraries across six treatments samples. Principal component analysis (PCA) of 18 libraries across six treatments samples. (A) PCA of 18 libraries. (B) PCA of six treatments samples. J12_(0–2): Three replicates of 2 days Lso-free; J17_(0–2): Three replicates of 7 days Lso-free; J32_(0–2): Three replicates of 2 days LsoB-infection; J37_(0–2): Three replicates of 7 days LsoB-infection; J42_(0–2): Three replicates of 2 days LsoA-infection; J47_(0–2): Three replicates of 7 days LsoA-infection.
(DOCX)

S2 Fig Cluster dendrogram of 18 libraries across six treatments samples. Cluster dendrogram of 18 libraries across six treatments samples. (A) Dendrogram of 18 libraries. (B) dendrogram of six treatments samples. J12_(0–2): Three replicates of 2 days Lso-free; J17_(0–2): Three replicates of 7 days Lso-free; J32_(0–2): Three replicates of 2 days LsoB-infection; J37_(0–2): Three replicates of 7 days LsoB-infection; J42_(0–2): Three replicates of 2 days LsoA-infection; J47_(0–2): Three replicates of 7 days LsoA-infection.
(DOCX)

S1 Table Primers for RT-qPCR validation. (DOCX)

S2 Table Summary statistics of transcriptome libraries. (DOCX)

S3 Table DEGs associated with zinc fingers. In objective1, for the LsoB category, the fold change values for the comparisons Lso-free vs. LsoB, and LsoB vs. LsoA are reported separated by the forward slash. Negative values indicate down-regulation in the first treatment.
(DOCX)

S4 Table Down-regulated DEGs in response to Lso. In objective1, for the LsoA category, the fold change values for the comparisons Lso-free vs. LsoA, and LsoB vs. LsoA are reported separated by the forward slash.; for the LsoB category, the fold change values for the comparisons Lso-free vs. LsoB, and LsoB vs. LsoA are indicated; for the LsoA&LsoB category, the fold change values for the comparisons Lso-free vs. LsoA, and Lso-free vs. LsoB are indicated. Negative values indicate down-regulation in the first treatment.
(DOCX)
