# Supplementary material for: Potato psyllids mount distinct gut responses against two different ‘Candidatus Liberibacter solanacearum’ haplotypes
Source: PLoS One. 2023 Jun 16;18(6):e0287396. doi: 10.1371/journal.pone.0287396 (PMC10275445; doi:10.1371/journal.pone.0287396)
Supplement: S2 Fig — Cluster dendrogram of 18 libraries across six treatments samples. (A) Dendrogram of 18 libraries. (B) dendrogram of six treatments samples. J12_(0–2): Three replicates of 2 days Lso-free; J17_(0–2): Three replicates of 7 days Lso-free; J32_(0–2): Three replicates of 2 days LsoB-infection; J37_(0–2): Three replicates of 7 days LsoB-infection; J42_(0–2): Three replicates of 2 days LsoA-infection; J47_(0–2): Three replicates of 7 days LsoA-infection. (DOCX) [file pone.0287396.s002.docx]

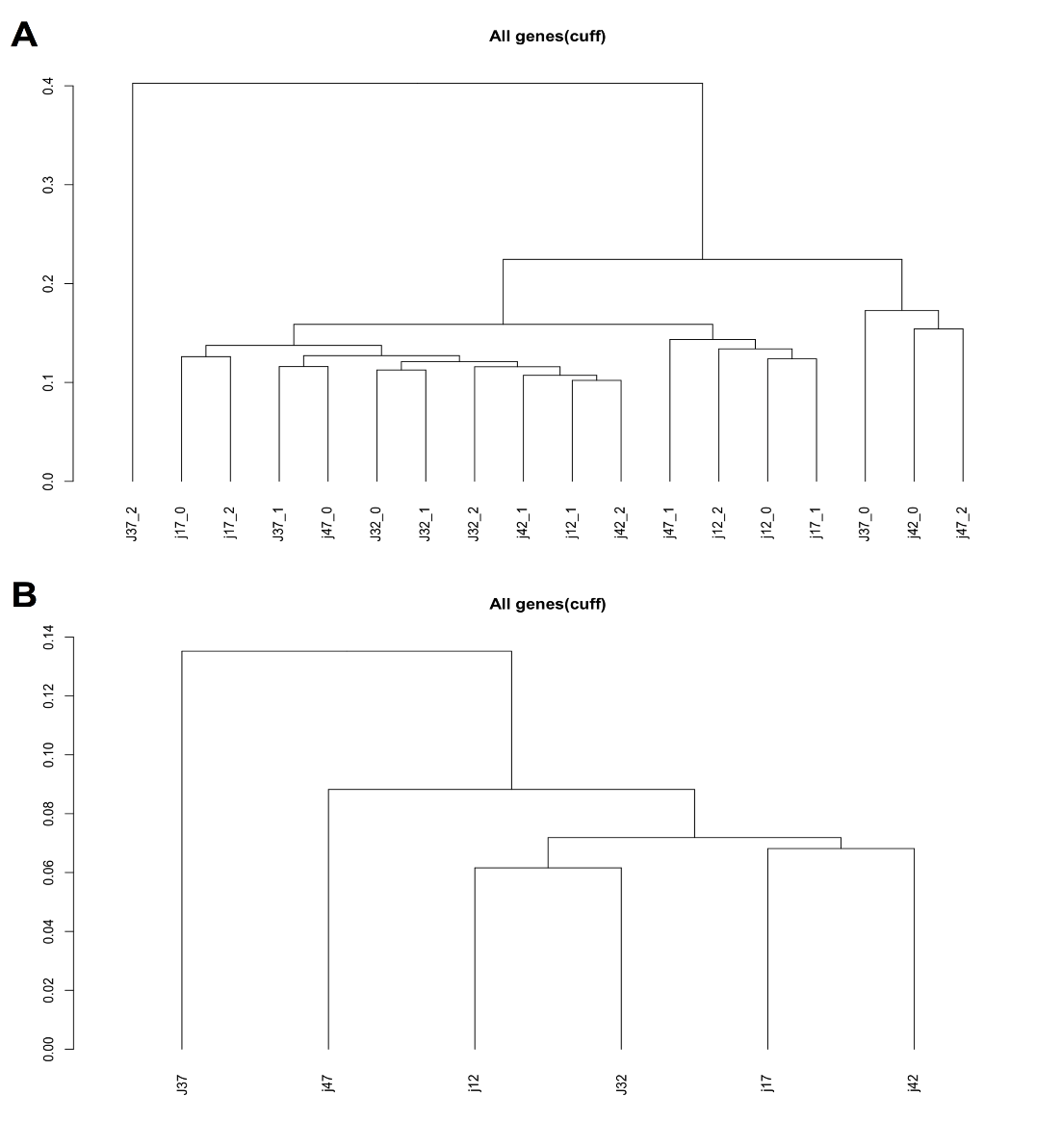


**Figure S2.** Cluster dendrogram of 18 libraries across six treatments samples. (A) Dendrogram of 18 libraries. (B) dendrogram of six treatments samples. J12_(0-2): three replicates of 2 days Lso-free; J17_(0-2): three replicates of 7 days Lso-free; J32_(0-2): three replicates of 2 days LsoB-infection; J37_(0-2): three replicates of 7 days LsoB-infection; J42_(0-2): three replicates of 2 days LsoA-infection; J47_(0-2): three replicates of 7 days LsoA-infection.
