# Supplementary material for: Potato psyllids mount distinct gut responses against two different ‘Candidatus Liberibacter solanacearum’ haplotypes
Source: PLoS One. 2023 Jun 16;18(6):e0287396. doi: 10.1371/journal.pone.0287396 (PMC10275445; doi:10.1371/journal.pone.0287396)
Supplement: S1 Table — (DOCX) [file pone.0287396.s003.docx]

**Table S1. Primers for RT-qPCR validation**

| Genes | Primers |
| --- | --- |
| Nedd4 | F: GCGCATCTTCTTCATAGACCA |
|  | R: CGGCTATTTGAGGATTGGAT |
| Hsp | F: TGAGAGAGATAGCCGAGGTG |
|  | R: TCTGGGAATCGTTGAAGTAGG |
| Patj | F: CACTACATCCGGTCCATCCT |
|  | R: GTGCTCATTCACCTCAAGCA |
| RASSF2 | F: TACGAGACAATGGCGAACAG |
|  | R: TGAGGACCCAGCATCACAC |
| HIGD2A | F: AGAAGGAACAGGATGAGTTGG |
|  | R: GAAGATCCACTCTCTGGGGTAA |
| Tret1 | F: CGTGTCTCATCCTGTTCGTC |
|  | R: ATCCCTTCCTCGTCCTCATT |
| UBE4A | F: TGAAACAACCTGGGCTCTATG |
|  | R: ACGATGTGGGAGAAGAAGGA |
| ZN728 | F: ACCGTATTGCTGCCACATCT |
|  | R: AAGGGTCTTTCGCCTGTATG |
| ZN91 | F: CGAGCGACTCAACAAACTGA |
|  | R: TGATGCAGTAGGGACACTGG |
| TRIP12 | F: GCAAGAGGTGCTAAAGAACGA |
|  | R: ATGAGGATCTCCGCCATCT |
