# Supplementary material for: Potato psyllids mount distinct gut responses against two different ‘Candidatus Liberibacter solanacearum’ haplotypes
Source: PLoS One. 2023 Jun 16;18(6):e0287396. doi: 10.1371/journal.pone.0287396 (PMC10275445; doi:10.1371/journal.pone.0287396)
Supplement: S2 Table — (DOCX) [file pone.0287396.s004.docx]

**Table S2. Summary statistics of transcriptome libraries**

| Samples | Reads number | Mapped reads | Mapped rate (%) |
| --- | --- | --- | --- |
| Lso-free 2-day rep1 | 46,373,575 | 19,940,637 | 43.0 |
| Lso-free 2-day rep2 | 49,049,463 | 21,630,813 | 44.1 |
| Lso-free 2-day rep3 | 42,454,067 | 18,000,524 | 42.4 |
| Lso-free 7-day rep1 | 40,597,962 | 16,117,391 | 39.7 |
| Lso-free 7-day rep2 | 47,157,120 | 20,088,933 | 42.6 |
| Lso-free 7-day rep3 | 39,518,942 | 16,479,399 | 41.7 |
| LsoA 2-day rep1 | 38,985,733 | 15,906,179 | 40.8 |
| LsoA 2-day rep2 | 40,627,811 | 16,291,752 | 40.1 |
| LsoA 2-day rep3 | 54,515,788 | 22,078,894 | 40.5 |
| LsoA 7-day rep1 | 48,360,594 | 20,118,007 | 41.6 |
| LsoA 7-day rep2 | 51,770,107 | 20,915,123 | 40.4 |
| LsoA 7-day rep3 | 47,470,121 | 18,655,758 | 39.3 |
| LsoB 2-day rep1 | 41,224,598 | 17,973,925 | 43.6 |
| LsoB 2-day rep2 | 38,599,255 | 15,902,893 | 41.2 |
| LsoB 2-day rep3 | 41,368,602 | 17,498,919 | 42.3 |
| LsoB 7-day rep1 | 36,623,405 | 15,857,934 | 43.3 |
| LsoB 7-day rep2 | 47,253,373 | 20,224,444 | 42.8 |
| LsoB 7-day rep3 | 46,148,068 | 19,059,152 | 41.3 |
