# Supplementary material for: Potato psyllids mount distinct gut responses against two different ‘Candidatus Liberibacter solanacearum’ haplotypes
Source: PLoS One. 2023 Jun 16;18(6):e0287396. doi: 10.1371/journal.pone.0287396 (PMC10275445; doi:10.1371/journal.pone.0287396)
Supplement: S3 Table — In objective1, for the LsoB category, the fold change values for the comparisons Lso-free vs. LsoB, and LsoB vs. LsoA are reported separated by the forward slash. Negative values indicate down-regulation in the first treatment. (DOCX) [file pone.0287396.s005.docx]

**Table S3. DEGs associated with zinc fingers**

|  | **GI number** | **Annotation** | **Species** | **Fold change** |
| --- | --- | --- | --- | --- |
| **Objective1: Haplotype** | **LsoB (7d)** |  |  |  |
|  | gi\|1041535729 | probable helicase with zinc finger domain | *Diaphorina citri* | 11.74/-6.96 |
|  | gi\|1041551763 | zinc finger protein 91-like | *Diaphorina citri* | 9.3/-7.77 |
|  | gi\|1062680758 | zinc finger CCHC domain-containing protein 8 homolog | *Drosophila navojoa* | 8.69/-7.74 |
|  | gi\|1041534625 | GATA zinc finger domain-containing protein 14-like | *Diaphorina citri* | 6.48/-6.31 |
|  | gi\|662220554 | zinc finger protein 64 homolog, isoforms 3 and 4-like | *Diaphorina citri* | 5.4/-4.4 |
|  | gi\|1041552204 | gastrula zinc finger protein XlCGF49.1-like, partial | *Diaphorina citri* | 6.08/-7.86 |
|  | gi\|1041545616 | zinc finger protein Xfin-like | *Diaphorina citri* | 6.91/-4.95 |
| **Objective2: Time** | **LsoA** |  |  |  |
|  | gi\|662200707 | AN1-type zinc finger protein 2A-like | *Diaphorina citri* | 2.87 |
|  | gi\|662193469 | zinc finger protein OZF-like | *Diaphorina citri* | 5.41 |
|  | gi\|662224129 | zinc finger protein 761-like | *Diaphorina citri* | 3.46 |
|  | gi\|1189056580 | AN1-type zinc finger protein 6 isoform X2 | *Drosophila serrata* | 3.13 |
|  | gi\|662220364 | zinc finger protein 91-like | *Diaphorina citri* | 3.07 |
|  | gi\|1041531946 | zinc finger protein 271 isoform X2 | *Diaphorina citri* | -6.05 |
|  | **LsoB** |  |  |  |
|  | gi\|1041532471 | zinc finger protein 271-like | *Diaphorina citri* | 14.39 |
|  | gi\|1041535729 | probable helicase with zinc finger domain | *Diaphorina citri* | 16.86 |
|  | gi\|1041535320 | zinc finger and BTB domain-containing protein 24-like | *Diaphorina citri* | 6.18 |
|  | gi\|1041545447 | zinc finger protein 64 homolog, isoforms 1 and 2-like | *Diaphorina citri* | 3.89 |
|  | gi\|662187921 | zinc finger protein 808-like | *Diaphorina citri* | 5.52 |
|  | gi\|1041545439 | zinc finger protein 711-like | *Diaphorina citri* | 4.61 |
|  | gi\|1041545447 | zinc finger protein 64 homolog, isoforms 1 and 2-like | *Diaphorina citri* | 7.8 |
|  | gi\|1041551763 | zinc finger protein 91-like | *Diaphorina citri* | 12.01 |
|  | gi\|1041533950 | zinc finger protein 84-like | *Diaphorina citri* | 4.65 |
|  | gi\|1041546623 | zinc finger protein 26-like | *Diaphorina citri* | 2.96 |
|  | gi\|1062680758 | zinc finger CCHC domain-containing protein 8 homolog | *Drosophila navojoa* | 5.97 |
|  | gi\|662188588 | zinc finger protein 728-like | *Diaphorina citri* | 5.09 |
|  | gi\|1041534625 | GATA zinc finger domain-containing protein 14-like | *Diaphorina citri* | 8.29 |
|  | gi\|1041545437 | zinc finger protein 107-like | *Diaphorina citri* | 5.17 |
|  | gi\|1041548843 | zinc finger protein OZF-like | *Diaphorina citri* | 10.33 |
|  | gi\|1041546007 | zinc finger protein 467-like | *Diaphorina citri* | 6.38 |
|  | gi\|1041548927 | zinc finger MYM-type protein 4-like | *Diaphorina citri* | 5.64 |
|  | gi\|1059385947 | zinc finger FYVE domain-containing protein 26 homolog isoform X2 | *Nicrophorus vespilloides* | 4.3 |
|  | gi\|1041533950 | zinc finger protein 84-like | *Diaphorina citri* | 6.31 |
|  | gi\|1041550280 | zinc finger protein 681-like | *Diaphorina citri* | 4.04 |
|  | gi\|1041537714 | zinc finger protein ZFAT-like | *Diaphorina citri* | 3.77 |
|  | gi\|662186483 | zinc finger protein OZF-like | *Diaphorina citri* | 4.51 |
|  | gi\|662188672 | zinc finger CCCH-type with G patch domain-containing protein | *Diaphorina citri* | 4.16 |
|  | gi\|1041533950 | zinc finger protein 84-like | *Diaphorina citri* | 4.15 |
|  | gi\|1060152124 | zinc finger matrin-type protein CG9776 isoform X2 | *Drosophila arizonae* | 5.43 |
|  | gi\|1101366102 | zinc finger SWIM domain-containing protein 5-like | *Bemisia tabaci* | 6.88 |
|  | gi\|1228358716 | centrosome-associated zinc finger protein CP190-like isoform X1 | *Folsomia candida* | 6.08 |
|  | gi\|961138841 | zinc finger protein 62 homolog | *Octopus bimaculoides* | 3.95 |
|  | gi\|1040686930 | gastrula zinc finger protein XlCGF57.1-like | *Danio rerio* | 3.38 |
|  | gi\|1041545449 | gastrula zinc finger protein XlCGF17.1-like | *Diaphorina citri* | 2.79 |
|  | gi\|662220554 | zinc finger protein 64 homolog, isoforms 3 and 4-like | *Diaphorina citri* | 7.61 |
|  | gi\|1041553783 | zinc finger protein 605-like | *Diaphorina citri* | 6.36 |
|  | gi\|662221164 | zinc finger protein 702 | *Diaphorina citri* | 5.36 |
|  | gi\|1041552204 | gastrula zinc finger protein XlCGF49.1-like | *Diaphorina citri* | 8.89 |
|  | gi\|1041545457 | zinc finger protein 513-like | *Diaphorina citri* | 5.13 |
|  | gi\|1041545457 | zinc finger protein 513-like | *Diaphorina citri* | 4.85 |
|  | gi\|1041530329 | zinc finger protein 64 homolog, isoforms 3 and 4-like | *Diaphorina citri* | 5.71 |
|  | gi\|662183487 | zinc finger MYND domain-containing protein 10 | *Diaphorina citri* | 6.67 |
|  | gi\|1041545616 | zinc finger protein Xfin-like | *Diaphorina citri* | 8.57 |
|  | **LsoA&LsoB** |  |  |  |
|  | gi\|1041533694 | zinc finger protein-like 1 homolog isoform X1 | *Diaphorina citri* | 3.34/3.82 |

In objective1, for LsoB, the value beside “/” indicates Lso-free vs. LsoB, and LsoB vs. LsoA, respectively; “-” indicates down-regulation.
