# Supplementary material for: Potato psyllids mount distinct gut responses against two different ‘Candidatus Liberibacter solanacearum’ haplotypes
Source: PLoS One. 2023 Jun 16;18(6):e0287396. doi: 10.1371/journal.pone.0287396 (PMC10275445; doi:10.1371/journal.pone.0287396)
Supplement: S4 Table — In objective1, for the LsoA category, the fold change values for the comparisons Lso-free vs. LsoA, and LsoB vs. LsoA are reported separated by the forward slash.; for the LsoB category, the fold change values for the comparisons Lso-free vs. LsoB, and LsoB vs. LsoA are indicated; for the LsoA&LsoB category, the fold change values for the comparisons Lso-free vs. LsoA, and Lso-free vs. LsoB are indicated. Negative values indicate down-regulation in the first treatment. (DOCX) [file pone.0287396.s006.docx]

**Table S4. Down-regulated DEGs in response to Lso**

|  | **GI number** | **Annotation** | **Species** | **Fold change** |
| --- | --- | --- | --- | --- |
| **Objective1: Haplotype** | **LsoB (2d)** |  |  |  |
|  | gi\|662205492 | pleckstrin homology domain-containing family J member 1-like | *Diaphorina citri* | -4.71/3.59 |
|  | gi\|662199817 | uncharacterized protein LOC103510220 | *Diaphorina citri* | -4.74/4.32 |
|  | gi\|1036730840 | alanine aminotransferase 1 | *Drosophila eugracilis* | -4.11/3 |
|  | gi\|662207585 | N-alpha-acetyltransferase 35, NatC auxiliary subunit-like | *Diaphorina citri* | -4.54/3.83 |
|  | gi\|662202860 | uncharacterized protein LOC103511784 isoform X1 | *Diaphorina citri* | -5.67/6.11 |
|  | gi\|662196843 | RNA pseudouridylate synthase domain-containing protein 3-like | *Diaphorina citri* | -10/4.42 |
|  | gi\|1060208525 | conserved oligomeric Golgi complex subunit 3 | *Drosophila arizonae* | -12/6.03 |
|  | gi\|662212760 | protein wntless | *Diaphorina citri* | -17.56/23.58 |
|  | **LsoA&LsoB (2d)** |  |  |  |
|  | gi\|1314956304 | beta-ureidopropionase | *Drosophila hydei* | -11.97/-12.29 |
|  | gi\|1041545896 | tudor domain-containing protein 1-like | *Diaphorina citri* | -9.92/-19.92 |
|  | **LsoA (7d)** |  |  |  |
|  | gi\|646706993 | Laminin subunit alpha | *Zootermopsis nevadensis* | -5.77/-5.34 |
|  | gi\|1041539257 | uncharacterized protein LOC103508912 | *Diaphorina citri* | -7.01/-12.64 |
|  | gi\|662204427 | probable small nuclear ribonucleoprotein E | *Diaphorina citri* | -3.17/-3.27 |
|  | **LsoB (7d)** |  |  |  |
|  | gi\|1101361217 | shootin-1 | *Bemisia tabaci* | -7.28/5.95 |
|  | gi\|662220628 | uncharacterized protein LOC103521131 | *Diaphorina citri* | -5.33/8.19 |
|  | **LsoA&LsoB (7d)** |  |  |  |
|  | gi\|1314956304 | beta-ureidopropionase | *Drosophila hydei* | -5.18/-8.6 |
|  | gi\|662196843 | RNA pseudouridylate synthase domain-containing protein 3-like | *Diaphorina citri* | -10.78/-8.34 |
| **Objective2: Time** | **LsoA** |  |  |  |
|  | gi\|1060228182 | laminin subunit alpha | *Drosophila arizonae* | -4.55 |
|  | gi\|1041539257 | uncharacterized protein LOC103508912 | *Diaphorina citri* | -8.26 |
|  | gi\|1041545157 | facilitated trehalose transporter Tret1-like | *Diaphorina citri* | -2.72 |
|  | gi\|1041547585 | galactokinase-like | *Diaphorina citri* | -2.94 |
|  | gi\|1036851719 | AP-3 complex subunit sigma-2 | *Drosophila ficusphila* | -3.28 |
|  | gi\|1101398038 | huntingtin | *Bemisia tabaci* | -4.31 |
|  | gi\|1189055661 | amidophosphoribosyltransferase | *Drosophila serrata* | -4.07 |
|  | gi\|1041539029 | uncharacterized protein LOC103508705 | *Diaphorina citri* | -3.77 |
|  | gi\|1041531946 | zinc finger protein 271 isoform X2 | *Diaphorina citri* | -6.05 |
|  | gi\|1041537311 | poly(rC)-binding protein 3-like | *Diaphorina citri* | -12.17 |
|  | **LsoB** |  |  |  |
|  | gi\|662190957 | 39S ribosomal protein L30, mitochondrial | *Diaphorina citri* | -3.57 |
|  | gi\|1036953778 | peptidyl-prolyl cis-trans isomerase-like 2 | *Drosophila bipectinata* | -2.83 |
|  | gi\|1041538717 | lipid storage droplets surface-binding protein 1-like | *Diaphorina citri* | -8.26 |
|  | gi\|1101361217 | shootin-1 | *Bemisia tabaci* | -14.75 |
|  | gi\|662219729 | ATP-binding cassette sub-family F member 1 | *Diaphorina citri* | -3.86 |

In objective1, for LsoA, the value beside “/” indicates Lso-free vs. LsoA, and LsoB vs. LsoA, respectively; for LsoB, the value beside “/” indicates Lso-free vs. LsoB, and LsoB vs. LsoA, respectively; for LsoA&LsoB, the value beside “/” indicates Lso-free vs. LsoA, and Lso-free vs. LsoB, respectively. “-” indicates down-regulation.
